# Molecular characterization of *bla*_*NDM*_*, bla*_*OXA-48*_*, mcr-1 and bla*_*TEM-52*_ positive and concurrently carbapenem and colistin resistant and extended spectrum beta-lactamase producing *Escherichia coli* in chicken in Malaysia

**DOI:** 10.1186/s12917-022-03292-7

**Published:** 2022-05-19

**Authors:** Erkihun Aklilu, Azian Harun, Kirnpal Kaur Banga Singh

**Affiliations:** 1 Faculty of Veterinary Medicine, Universiti Malaysia Kelantan, Locked Box 36, Pengkalan Chepa, 16100 Kota Bharu, Kelantan Malaysia; 2 Department of Medical Microbiology and Parasitology, School of Medical Sciences, Universiti Sains Malaysia, 16150 Kubang Kerian, Kota Bharu, Kelantan Malaysia

**Keywords:** *Escherichia coli*, ESBL, CRE, Antimicrobial resistance, Food animals, Public health, Food safety

## Abstract

**Background:**

Antimicrobial resistance (AMR) is a global public health threat and the use of antibiotics growth promoters in food animals has been implicated as a potential contributing factor in the emergence and spread of AMR. This study was conducted to investigate colistin and carbapenem resistance and extended spectrum beta-lactamase producing *E. coli* from live broiler chicken and chicken meat in Kelantan, Malaysia.

**Results:**

Among the *E. coli* isolates, 37.5% (27/72 were positive for at least one of the resistance genes and one isolate was positive for *mcr*-1, *bla*_TEM-52_, *bla*_*NDM*_ and *bla*_*OXA-48*_ whereas 4.17% (3/72) and 2.78% (2/72) were positive for *mcr*-1, *bla*_TEM-52_ and *bla*_*OXA*-48_, and mcr-1, *bla*_*TEM-52*_ and *bla*_*IMP*_. Multilocus sequence typing (MLST) results revealed the presence of widespread *E. coli* strains belonging to the sequence types ST410 and ST155 and other extra-intestinal *E. coli* (ExPEC) strains. Phylogroup A made up the majority 51.85% (14/27) followed by phylogroup B1 22.22% (6/27).

**Conclusions:**

The findings imply the potential threats of colistin, extended-spectrum beta-lactamase producing and carbapenem resistant *E. coli* in food animals to the public health and underscores the need for judicious use of antibiotics in animal production and good hygiene practices to curb the rising risks of AMR.

## Introduction

Antimicrobial resistance (AMR) represents a growing threat as an emerging worldwide problem in global health including medical science, veterinary and agricultural fields. Many microorganisms that were previously sensitive to antimicrobial agents continue to develop resistance to several antimicrobial agents [1]. The dangers of antimicrobial resistant bacteria lie in the possibilities of mild infections proving lethal and ultimately making the use of specific antibiotics towards resistant bacteria irrelevant [2].

The rise of antimicrobial resistance in food borne pathogens along with the increased use of antibiotics in food animal production have further compounded the threats of antimicrobial resistance worldwide, mainly in developing countries [3]. *Escherichia coli* is among the most prevalent etiological agents that causes several diseases of economic significance in poultry. These economic losses are related to high mortality, poor weight gain of infected chicken and poor carcass quality. Antibiotics have been used to control disease outbreaks including infections such as colibacillosis, thus reducing morbidity and mortality rates [4]. Even though antibiotics are highly recommended for therapeutic purpose, certain class of antibiotics have also been used widely in sub-therapeutic doses in food producing animals as growth promoters and/or to prevent occurrence of disease outbreaks in farms which in turn has been contributing to enhanced selection of resistant bacteria in livestock [5]. This poses serious challenge to the public health since antibiotic resistance in human can be acquired through the food chain [6]. Several studies have attributed the emergence and spread of multidrug resistant *E. coli* in food animals to the irrational use of antibiotics thereby reducing the effectiveness of antimicrobial agent that are commonly used both in medical and veterinary practices [7]. To curb the rising problems of AMR in food animals, several countries, mostly developed counties have banned the use of antibiotic growth promoters in animal production. Colistin, a polymyxin class of antibiotic has been considered as the last resort antibiotics to combat bacteria that are resistant to other potent and broad-spectrum antibiotics in clinical uses. The emergence of multidrug-resistant Gram-negative bacteria has led to the reintroduction of colistin as a last resort antibiotic for the treatment of severe infections [8]. In contrast to its limited use in humans because of reported high incidence of nephrotoxicity and neurotoxicity, colistin has been widely used in food-producing animals in many countries. Colistin sulphate possess an excellent activity against *E. coli* and *Salmonella enterica* and is commonly used in oral preparation in veterinary settings. In poultry and pig production, colistin has been said to have low frequency of resistance [9]. However, latest discovery revealed that colistin may be losing its clinical efficacy in antimicrobial therapy as Gram-negative bacteria like *E. coli* have mutated to become resistant to this last resort antibiotics [10]. The possibilities of spread of colistin resistance through horizontal gene transfer contributes to the spread of resistance among closely related bacteria, Enterobacterales family, including *E. coli*. Recent studies conducted in Kelantan, Malaysia reported that chicken meat sold in local market in Kelantan are contaminated with *E. coli* which were resistant to colistin and positive for *mcr-1* and *bla*_TEM-52_ and *bla*_CTX_ genes [11, 12].

Antimicrobial resistance among *E. coli* isolates has been increasing and contributing to the challenges and complexity in management of extraintestinal infections in humans [13]. Prevention and control of the spread of colistin resistant *E. coli* causing infections requires an understanding of the population genetics of this pathogen. Recent studies have demonstrated that *E. coli* population is largely clonal and strains fall into four major phylogroups (A, B1, B2 and D). Several reports have suggested poultry as a reservoir for multidrug resistant *E. coli* strains causing infections in humans [14, 15]. Investigation of colistin resistance, extended beta-lactamase production and carbapenem resistance in *E. coli* isolates from live chicken and chicken meat can provide useful data for the evaluation of these multidrug resistant pathogens’ zoonotic potentials and devise applicable control and prevention strategies based on local context and global epidemiology of the pathogens. This study was conducted to investigate the presence of extended spectrum beta-lactamase positive, colistin and carbapenem resistant *E. coli* from live chicken and raw chicken meat in Kelantan, Malaysia and to determine the phylogenetic diversity of the *E. coli* isolates to better understand their distribution and characteristics. The presence of resistance genes, *bla*_*TEM-52*_, *bla*_*CTX*_, *bla*_*OXA-48*_, *bla*_*NDM*_, *bla*_*IMP*_ genes in the *E. coli* isolates were investigated.

## Materials and methods

### Phenotypic and molecular identification of *E. coli*

One hundred and twenty cloacal swabs from four weeks old broiler chickens from four different farms and 100 raw chicken meat samples were collected from five different local outlets in Kelantan, Malaysia were analyzed. The sample size was calculated based on 95% confidence level, 5% margin of error and 11% prevalence [16]. Chicken meat samples (breast, wing, thigh, and keel) were aseptically collected from supermarket outlets. The five outlets were selected based on convenience and within the Kota Bharu district of Kelantan state. Other outlets including wet markets were and chicken meat with grossly visible contamination were excluded. Aseptic techniques were followed during sample collection, storage, and processing. Only four weeks or older broiler chickens from four farms were included in the sampling. Whereas chicken younger than four weeks old, layers and breeder farms and chicken with any clinical signs of illness were excluded from the sampling. The cloacal swabs were collected using a swab with Amies transport media (Oxoid, UK) and the meat samples were collected in sterile zip lock bag containing 0.90% w/v of NaCl. The samples were either processed on the same day or stored in at 4 °C overnight for next day processing. Both the processed chicken meat and swab samples were then inoculated into LB Broth and aerobically incubated at 37 °C overnight for enrichment. Routine bacteria isolation and identification was done using differential and selective growths of *E. coli* using MacConkey, Nutrient, Eosine Methylene Blue (EMB) agars (Oxoid, UK), Gram staining and biochemical tests. Presumptive *E. coli* isolates were further confirmed using PCR amplification of *E. coli* specific gene, *pho* using primer sets as in Table 1.

**Table 1:** Sequence of the oligonucleotides used for detection of resistance genes and phylotyping of *E. coli *isolates

Primers	Sequence (5’–3’)	Product size (bp)	Target gene	References
Pho-F	GTGACAAAAGCCCGGACACCATAAATGC	903	*pho*	[17]
Pho-R	TACACTGTCATTACGTTGCGGATTTGGCG			
MCR1-F2	AGTCCGTTTGTTCTTGTGGC	320	*mcr-1*	[18]
MCR1-R2	AGATCCTTGGTCTCGGCTTG			
MCR2-F	CAAGTGTGTTGGTCGCAGTT	715	*mcr-2*	
MCR2-R	TCTAGCCCGACAAGCATACC			
MCR3-F	AAATAAAAATTGTTCCGCTTATG	929	*mcr-3*	
MCR3-R	AATGGAGATCCCCGTTTTT			
MCR4-F	TCACTTTCATCACTGCGTTG	1116	*mcr-4*	
MCR4-R	TTGGTCCATGACTACCAATG			
MCR5-F	ATGCGGTTGTCTGCATTTATC	1644	*mcr-5*	
MCR5-R	TCATTGTGGTTGTCCTTTTCTG			
blaTEM-52 F	ATAAAATTCTTGAAGACGAAA	1080	*bla*_*TEM*_	[19]
blaTEM-52 R	GACAGTTACCAATGCTTAATC			
blaCTX-F	CCCATGGTTAAAAAACACTGC	950	bla_CTX_	[20]
blaCTX-R	CAGCGCTTTTGCCGTCTAAG			
NDM-F	GGTTTGGCGATCTGGTTTTC	621	*bla*_*NDM*_	[21]
NDM-R	CGGAATGGCTCATCACGATC			
IMP-F	GGAATAGAGTGGCTTAAYTCTC	232	*bla*_*IMP*_	
IMP-R	GGTTTAAYAAAACAACCACC			
OXA-F	GCGTGGTTAAGGATGAACAC	438	*bla*_OXA-48_	
OXA-R	CATCAAGTTCAACCCAACCG			
ChuA.1	GAC GAA CCA ACG GTC AGG AT	279	*chu*A	[22]
ChuA.2	TGC CGC CAG TAC CAA AGA CA			
YjaA.1	TGA AGT GTC AGG AGA CGC TG	211	*yja*A	
YjaA.2	ATG GAG AAT GCG TTC CTC AAC			
TspE4.C2.1 TspE4.C2.2	GAG TAA TGT CGG GGC ATT CA CGC GCC AAC AAA GTA TTA CG	152	*Tsp*E4.C2	
AceK.f	AACGCTATTCGCCAGCTTGC	400	*arpA*	
ArpA1.r	TCTCCCCATACCGTACGCTA			
ArpAgpE.f	GATTCCATCTTGTCAAAATATGCC	301	*arpA*	
ArpAgpE.r	GAAAAGAAAAAGAATTCCCAAGAG			
trpAgpC.1	AGTTTTATGCCCAGTGCGAG	219	*trpA*	
trpAgpC.2	TCTGCGCCGGTCACGCCC-			
trpBA.f	CGGCGATAAAGACATCTTCAC	489	*trpA*	
trpBA.r	GCAACGCGGCCTGGCGGAAG			

### Antibiotic susceptibility testing

The disk diffusion method was used to determine the susceptibility of all the detected *E. coli* isolates to several antibiotics. The bacterial colony from nutrient agar was used to make a suspension in sterile 0.9% NaCl to 0.5 McFarland standard and was spread on Mueller–Hinton agar (Oxoid, UK). Ten different antibiotics that are typically used for treatment of infections caused by *E. coli* were selected based on the OIE List of Antimicrobial Agents of Veterinary Importance 2014. The antibiotics with the following concentrations were used in this study: erythromycin (15 µg), tetracycline (30 µg), colistin (10 µg), ceftioufur (30 µg), polymyxin B (300 µg), amoxycilin (30 µg), ampicillin (25 mg), gentamicin (10 µg), sulphafurazole (300 mg), and cephalothin (5 mg), trimethoprim (5 µg), streptomycin (10 µg). After aerobic incubation for 24 h at 37 °C, the organisms were classified as sensitive or resistant according to the inhibition zone diameter. Zones of inhibition were measured to the nearest millimeter and were reported either as sensitive (S), intermediate resistant (I) or resistant (R) according to the guidelines of the Clinical Standard Laboratory Institute [23].

### Carbapenem inactivation method

Carbapenem inactivation method was used for phenotypic detection of carbapenem resistance as recommended by the Clinical Laboratory Standard Institute [23]. Briefly, a 1µL loopful of *E. coli* from an overnight blood agar plate was emulsified in 2 mL TSB and was vortexed for 10–15 s. A 10-µg meropenem disk was placed in each tube using sterile forceps ensuring that the entire disk is immersed in the suspension. The suspension was incubated at 35 °C in ambient air for 4 h. A 0.5 McFarland suspension (using the colony suspension method) of *E. coli* ATCC® 25,922 was prepared in nutrient broth and was inoculated on the MHA plate making sure that the inoculum suspension preparation and MHA plate inoculation steps are each completed within 15 min. The plates were allowed to dry for 3–10 min before adding the meropenem disks. The meropenem disk from each TSB-meropenem disk suspension was removed using a 10-µL loop and was placed on the flat side of the loop against the flat edge of the disk and using surface tension to pull the disk out of the liquid. The disk was carefully dragged while pressing the loop along the inside edge of the tube to expel excess liquid from the disk and was placed on the MHA plate previously inoculated with the meropenem-susceptible *E. coli* ATCC® 25,922 indicator strain. The plates were inverted and incubated at 35 °C in ambient air for 18–24 h. Following incubation, the zones of inhibition measure were measured and interpreted according to CLSI guidelines [23].

### Colistin broth disk elution test

To determine the minimum inhibitory concentration (MIC) values for colistin, a CLSI recommended colistin broth disk elution [23] was conducted. Briefly, a 10-μg colistin disks making a final concentration of 0 μg/mL (growth control), 1 μg/mL, 2 μg/mL, and 4 μg/mL colistin were prepared. Using a loop 3–5 colonies from a fresh (18–24 hours) nutrient agar culture was transferred to sterile saline (4–5 mL). The cation-adjusted Muller-Hinton Broth (CAMHB) tubes (10 mL) and colistin disks were warmed to room temperature. Four tubes of CAMHB for each isolate to be tested with 1, 2, and 4 µg/mL and control were prepared. Using aseptic technique, 1 colistin disk to the tube labelled “1 µg/mL”, 2 colistin disks to tube labelled “2 µg/mL”, and 4 colistin disks to the tube labelled “4 µg/mL” were carefully added to separate tubes. The tubes were gently vortexed and the colistin was allowed to elute from the disks for 45 minutes at room temperature. A 0.5 McFarland standard of the bacterial test inoculums were prepared and 50µL of the standardized inoculum were added to the control and 1-, 2-, and 4-µg/mL tubes to attain a final inoculum concentration of approximately 7.5 × 105 CFU/mL. Ten microlitre from the original inoculum tube was inoculated on blood agar plate for a purity check. The tubes were tightly capped and each inoculated tube was vortexed on slow speed to mix. The slow speed is suggested to prevent colistin from sticking to the cap and glass surface above the meniscus of liquid. The caps were slightly loosened before incubation and incubated at 35°C in ambient air for 16–20 hour. The purity plates were examined to ensure inoculum was pure. The growth control tube was also examined to ensure the presence of obvious turbidity for the test to be valid. The MIC as the lowest concentration that completely inhibits growth of the test isolate was determined using the following cut-off points for Enterobacterales and *P. aeruginosa (*≤ 2 μg/mL = intermediate ≥ 4 μg/mL = resistant). *E. coli *ATCC® 25922 was used as control. 

### ESBL production test

Phenotypic confirmatory tests of the *bla*_*TEM-52*_ positive *E. coli* isolates were done by using Modified Double Disk Synergy Test (MDDST) as described earlier [24]. Amoxicillin-clavulanate (20/10 μg) along with four cephalosporins; 3GC-cefotaxime, ceftriaxone, cefpodoxime and 4GC-cefepime were used to conduct the MDDST. The test isolates were suspended in 0.9% NaCl solution and the inoculum concentration was adjusted to 0.5 McFarland standard. The inoculum was lawn on Muller-Hinton agar (OXOID, UK) and the plates were allowed to dry for about 10 min. Amoxicillin-clavulanate (20/10 μg) was placed in the centre of the plate. The 3GC and 4GC disks were placed 15 mm and 20 mm apart respectively, centre to centre to that of the amoxicillin-clavulanate disk. Any distortion or increase in the inhibition zone towards the amoxicillin-clavulanate disk was considered as positive for the ESBL production. *Escherichia coli* 25,922 was used as a negative control for the ESBL production.

### Determination of minimum inhibitory concentration by Etest

The minimum inhibitory concentrations (MIC) for carbapenems (meropenem) and extended spectrum beta lactams (Cefotaxime) as additional phenotypic characterization of carbapenem resistant and extended spectrum beta-lactamase producing *E. coli* was conducted. The MIC values were determined by using Etest (Biomerieux, USA) following the manufacturer’s instructions. The E. coli isolates were grown over night on nutrient agar and 2–3 colonies were taken and suspended in a 0.9% NaCl solution and the turbidity was adjusted to 0.5 McFarland standard. The suspension was uniformly inoculated on Muller-Hinton agar (OXOID, UK) using sterile swab and was left for about 5 min to allow drying. The Etest strips were carefully placed on the inoculated plate using sterile forceps and a gentle pressure was applied on the strips to ensure adhesion of the strips on the inoculated media. The inoculated plates were incubated at 37 °C aerobically for 24 h. *E. coli* ATCC® 25,922 was used as control. The results were interpreted using the CLSI guidelines [23].

### Amplification of Colistin resistance (mcr1-mcr5), Extended spectrum beta-lactamse (bla_CTX_ and bla_TEM-52_ and Carbapenemase (bla_NDM_, bla_OXA-48_ and bla_IMP_) genes

Genomic DNA was extracted from overnight cultures of *E. coli* grown in Brain Heart Infusion agar (BHIA) (Oxoid, UK) using DNA, RNA, and Protein purification kit (Macherey–Nagel, Germany) following the manufacturer’s recommendations. Three different multiplex PCR sets comprising SetA (*mcr*-1, *mcr*-2, *mcr*-3, *mcr*-4 and *mcr*-5), SetB (*bla*_CTX-M_ group, and *bla*_TEM-52_) and SetC (*bla*_*NDM*_*, bla*_*OXA-48*_* and bla*_*IMP*_) were separately run and single PCR amplification of each gene were also conducted for further confirmation. Each set of multiplex PCR was prepared in a total 50 µL volume comprising 5 µL of bacterial DNA was added into 25 µL of 2 × Taq Mastermix (Promega, USA), 1 µL (10 µm) of each primer (Table 1) and nuclease free water. The multiplex PCR was conducted using the following protocols. SetA: initial denaturation at 94 °C for 3 min, followed by 30 cycles of denaturation at 94 °C for 45 s, annealing at 60 °C for 1 min, extension at 72 °C for 3 min and final extension at 72 °C for 5 min. SetB: initial denaturation at 94 °C for 4 min followed by 30 cycles of denaturation at 94 °C for 1 min, annealing at 54 °C for 1 min, extension at 72 °C for 2 min and final extension at 72 °C for 5 min. SetC: initial denaturation at 94 °C for 3 min followed by 36 cycles of denaturation at 94 °C for 45 s, annealing at 56 °C for 1 min, extension at 72 °C and final extension at 72 °C for 5 min. *Escherichia coli* ATCC25922 strain was used as control.

### Determination of phylogenetic diversity

Phylogenetic classification of *E. coli* isolates was performed using the revised Clermont method PCR method [22]. The primer sequences pairs used for PCR amplification are shown in the Table 1. Multiplex PCR reaction was performed in a 25μL reaction mixture, containing reaction buffer (pH 8.5), 400 µM dATP, 400 µM dGTP, 400 µM dCTP, 400 µM dTTP and 3 mM MgCl_2_, each primer (1 μM), Taq DNA polymerase (1U), and template DNA (5μL). Negative controls (reaction without template DNA) and a positive control were included in all performed amplifications. The PCR reaction began with initial denaturation at 94 °C for 4 min, 30 cycles of 5 s at 94 °C and 10 s at 57 °C, and a final extension step at 72 °C for 5 min. *Escherichia coli* ATCC25922 strain was used as control. All the PCR products were analyzed in 1.5% agarose gel using gel electrophoresis set at 100 V and 400 mA for 40 min. The gel images were viewed and analyzed using GelDoc® (BIO-RAD, USA).

### Multilocus sequence typing

The multilocus sequence typing of selected isolates (CS1, CS2, CS3, CS4, CS5, CS6, CS8, CS9, CM1, CM3, CM6, CM9) selected based on resistance profiles were determined as described previously [25]. The PCR amplification and sequencing of the seven housekeeping genes (*adk*, *fum*C, *gyr*B, *icd*, *mdh*, *pur*A and *rec*A) were performed following the protocols recommended for *E. coli* (http://mlst.warwick.ac.uk/mlst/dbs/Ecoli). The primer sequences of all seven genes are available at https://enterobase.readthedocs.io/en/latest/mlst/mlst-legacy-info-ecoli.html. The PCR reactions were prepared in a 50 μL amplification reaction mixture comprised of 5 μL of template DNA, 1 μL of each primer (25pmol/μL), 25 μL GoTaq^®^ Master Mix (Promega, USA) and 18 μL sterilized distilled water. The amplification conditions were set at an initial denaturation step at 94°C for 2 min, followed by 30 cycles of the following conditions: denaturation at 94°C for 1 min, 1 min primer annealing at 54–60°C, and extension at 72°C for 2 min, with a final extension step at 72°C for 5 min.

## Results

### Detection of carbapenem and colistin resistance and beta-lactamase genes

Out of the total 220 samples (120 cloacal and 100 raw chicken), 72 (32.73%) PCR confirmed *E. coli* were detected. Twenty-three of these isolates were from raw chicken meat while the remaining 49 were from cloacal swabs. From these, 37.5% (27/72) of the *E. coli* isolates were positive for at least one of the resistance genes. One isolate was positive for mcr-1, *bla*_*TEM-52*_, *bla*_*NDM*_ and *bla*_*OXA*-48_ whereas 4.17% (3/72), 2.78% (2/72), 1.39% (1/72), 11.11% (8/72) and 4.17% (3/72) were positive for *mcr*-1, *bla*_TEM-52_ and *bla*_*OXA*-48_, mcr-1, *bla*_TEM-52_ and bla_*IMP*_, mcr-1, *bla*_TEM-52_ and *bla*_*NDM*_, *mcr*-1 and *bla*_TEM-52_, and *bla*TEM-52 and *OXA*-48 respectively (Table 2). However, none of the isolates were positive for *mcr*-2, *mcr*-3, *mcr*-4, *mcr*-5 or *bla*_CTX_.

**Table 2:** Phenotypic and genotypic characteristics of carbapenem and colistin resistant and ESBL producing *E. coli* isolates from cloacal swabs and chicken meat

**Isolate ID**	**Source**	**Antibiotics resistance (disk diffusion)**	**MIC Values** (µg/mL)			**Resistance genes detected**	**Phylogenetic group**	**MLST** **(ST)**
			**Meropenem (Etest)**	**Colistin**	**Cefotaxime (Etest)**			
CS1	*Cloacal swab*	CAZ30, W5, AML10, CT10, TE30, C30, SC300, ENR5	2	≥ 4	4	*mcr*-1, *bla*_*TEM-52*_ and *bla*_*OXA-48*_	A	155
CS2	*Cloacal swab*	CAZ30, AML10, C30, SC300, CN10, ENR5	2	≥ 4	8	*mcr*-1, *bla*_*TEM-52*_ and *bla*_*OXA-48*_	A	2179
CS3	*Cloacal swab*	CAZ30, IPM10, W5, CT10, TE30, C30, SC300	2	≥ 4	4	*mcr*-1, *bla*_*TEM-52*_ and bla_*OXA*-48_	Unknown	872
CS4	*Cloacal swab*	CAZ30, W5, CT10, TE30, C30, SC300, ENR5	8	≥ 4	16	*mcr*-1, *bla*_*TEM-52*_ and *bla*_*NDM*_	B1	410
CS5	*Cloacal swab*	CAZ30, W5, AML10, CT10, TE30, C30, ENR5	8	≤ 2	2	*bla*_*OXA*-48_ and bla_*NDM*_	A	2179
CS6	*Cloacal swab*	CAZ30, IPM10, W5, AML10, CT10, TE30, C30, SC300, CN10	16	≥ 4	4	*mcr*-1, *bla*_TEM-52_, *bla*_*NDM*_ and *bla*_*OXA*-48_	A	410
CS7	*Cloacal swab*	CAZ30, W5, AML10, CT10, TE30, C30, ENR5	0.5	≥ 4	0.5	*mcr*-1	B2	-
CS8	*Cloacal swab*	CAZ30, IPM10, TE30, C30, SC300	0.25	≤ 2	2	*mcr*-1, *bla*_TEM-52_ and *bla*_*IMP*_	B1	373
CS9	*Cloacal swab*	CAZ30, IPM10, W5, CT10, TE30, SC300	0.5	≥ 4	4	*mcr*-1, *bla*_*TEM-52*_ and *bla*_*IMP*_	A	155
CS10	*Cloacal swab*	CAZ30, W5, AML10, CN10	0.5	≤ 2	0.025	*bla*_*TEM-52*_	A	-
CS11	*Cloacal swab*	CAZ30, W5, AML10	0.25	≤ 2	0.25	*bla*_*TEM-52*_	A	-
CS12	*Cloacal swab*	CAZ30, AML10	0.5	≥ 4	0.5	*mcr*-1	Unknown	-
CM1	*Chicken meat*	CAZ30, W5, CT10, CT10, TE30, SC300	0.25	≤ 2	4	*bla*_OXA-48_ and *bla*_*IMP*_	A	168
CM2	*Chicken meat*	W5, AML10, ENR5	0.25	≥ 4	0.25	*mcr*-1 only	A	-
CM3	*Chicken meat*	CAZ30, IPM10, SC300, ENR5	0.25	≤ 2	4	*mcr-*1 and *bla*_*TEM-52*_	A	770
CM4	*Chicken meat*	CAZ30, AML10, ENR5	0.5	≥ 4	0.025	*mcr-*1 and *bla*_*TEM-52*_	A	-
CM5	*Chicken meat*	CAZ30, W5, CT10, TE30, C30, SC300	0.05	≥ 4	0.25	*mcr-*1 and *bla*_*TEM-52*_	A	-
CM6	*Chicken meat*	W5, AML10, CT10, TE30, SC300, ENR5	0.25	≥ 4	4	*bla*_*TEM-52*_ and bla_OXA-48_	D	6588
CM7	*Chicken meat*	CAZ30, AML10, TE30, C30, SC300	0.5	≥ 4	0.5	*mcr-*1 and *bla*_*TEM-52*_	B1	-
CM8	*Chicken meat*	CAZ30, W5, ENR5	0.25	≤ 2	0.25	*mcr-*1 and *bla*_*TEM-52*_	A	-
CM9	*Chicken meat*	CAZ30, CT10, TE30, C30, SC300, ENR5	0.25	≥ 4	4	*bla*_*TEM-52*_ and bla_OXA-48_	B1	2058
CM10	*Chicken meat*	AML10, CT10, TE30, C30, SC300	0.5	≤ 2	0.25	*mcr-*1 and *bla*_*TEM-52*_	Unknown	-
CM11	*Chicken meat*	CAZ30, CT10, TE30, C30, SC300	0.5	≤ 2	0.5	*mcr-*1 and *bla*_*TEM-52*_	A	**-**
CM12	Chicken meat	AML10, CT10, TE30, C30, CN10, ENR5	0.5	≤ 2	0.25	*bla*_*TEM-52*_ and *bla*_*OXA-48*_	B1	**-**
CM13	Chicken meat	W5, AML10, ENR5	0.25	≥ 4	0.025	*mcr*-1	D	**-**
CM14	Chicken meat	AML10, CN10, ENR5	0.25	≥ 4	0.25	*bla*_*TEM-52*_	F	**-**
CM15	Chicken meat	W5, AML10, ENR5	0.25	≥ 4	0.25	*mcr-*1 and *bla*_*TEM-52*_	B1	**-**

### Phenotypic confirmation of resistance

The phenotypic characterization of resistance towards carbapenems, colistin and extended spectrum beta-lactamase showed that some of the isolates which harbored the resistance genes did not show resistance phenotypically. All the carbapenem resistant *E. coli* isolates that were positive for either *bla*_*NDM*_ or *bla*_*OXA-48*_ or both genes (*n* = 9) were also shown to be carbapenem resistant when tested using carbapenem inactivation method. However, only one isolate out of three which were positive for IMP gene showed phenotypic resistance. Likewise, nearly half or 44.44% (12/21) of the isolates that were positive for *bla*_*TEM-52*_ gene did not show phenotypic resistance either by Etest or MDDST. Similar pattern has been observed for the phenotypic confirmation of colistin resistance by colistin broth disk elution method where 14.29% (3/21) of the mcr1-positive isolates did not show phenotypic resistance.

### Antimicrobial susceptibility testing

Most of the colistin resistant *E. coli* show resistance towards sulphonamide, tetracycline, doxycycline followed by chloramphenicol, colistin, trimetoprim-sulfametoxazol, cefazolin and enrofloxacin. The antimicrobial susceptibility testing results showed that all *E. coli* isolates from different phylogroups were susceptible to imipenem and gentamycin antibiotics except for three isolates from group A which were resistance to the antibiotic. *Escherichia coli* isolates belonging to phylogenetic groups A and B1 showed different susceptibility levels to colistin compared to *E. coli* isolates from phylogroup D. High prevalence of multidrug resistance *E. coli* isolates were mainly detected in group B1 which indicates that strains belonging to phylogroups A and B1 carried more resistance properties than the other phylogroups. Multidrug resistant *E. coli* was detected with resistance ranging from 2 to 10 antibiotics tested. The overall AST results of the *E. coli* isolates were as in Table 2.

### Sequence types and phylogenetic grouping

A total of 27 *E. coli* strains positive for the resistance genes were assigned to four phylogroups (A, B1, B2, D, F and Unknown). According to the multiplex PCR-based phylotyping, group A comprised most of the collected isolates 51.85% (14/27) followed by phylogroup B1 22.22% (6/27). Other identified phylogroups were B2, D, F, and Unknown group. Seven isolates of group D were assigned to subgroup D2. The analysis profile using multiplex PCR was shown in Table 2.

## Discussions

Infections caused by *E. coli* species have been dramatically increasing worldwide and constitute significant public health risks. In addition to causing several animal diseases and significant economic losses in food animal production industry, pathogenic *E. coli* species also cause several life-threatening diseases in humans [14, 26]. This study revealed that 37.5% (27/72) of the *E. coli* isolated from 120 samples were positive for at least one of the resistance genes *bla*_*NDM*_, *bla*_*OXA-48*_, *mcr*-1, and *bla*_*TEM-52*_. One isolate was positive for *mcr*-1, *bla*_*TEM-52*_, *bla*_*NDM*_ and bla_*OXA*-48_ marking the multidrug resistance of the isolate against colistin, extended spectrum beta-lactams and carbapenem antibiotics. Whereas 4.17% (3/72) positive for *mcr*-1, *bla*_*TEM-52*_ and *bla*_*OXA*-48_, 2.78% (2/72) for *mcr-1*, *bla*_*TEM-52*_ and *bla*_*IMP*_, 1.39% (1/72) for mcr-1, *bla*_*TEM-52*_ and *bla*_*NDM*_, 11.11% (8/72) for mcr-1 and *bla*_*TEM-52*_ and 4.17% (3/72) for *bla*_*TEM-52*_ and *bla*_*OXA*-48_ genes. However, the results from phenotypic resistance detection methods showed that some of the isolates which were positive for one or more of the resistance genes *bla*_*IMP*_, *bla*_*TEM-52*_ and *mcr-1* did not show evidence of resistance towards the respective antibiotics tested. These results show discrepancies when compared with the results from genotypic (PCR) detection of resistance implying the fact that presence of a resistance gene does not necessarily confirm phenotypic resistance. Thus, the CLSI recommends the phenotypic detection methods for routine and clinical confirmation of carbapenem resistance, resistance to extended spectrum beta lactams and colistin resistance [23]. Nevertheless, PCR-based methods have been widely accepted as gold standard for confirmation of resistance and are rapid, robust and reliable in detecting most resistant bacteria including carbapenemases [27]. A recent study by Jochum et al. [28] reported a wide-spread abundance of carbapenem resistant *E. coli* (CRE) in commercial chicken farms in USA. The same study also reported the presence of extraintestinal pathogenic *E. coli* phylogenetic groups B2 and D from the chickens. Another study from Bangladesh reported 13.5% (14/104) *mcr*-1 positive *E. coli* which were also resistant to third-generation cephalosporin of which two isolates carried additional resistant genes including *bla*_CTX-M_ group-1 and *bla*_*TEM-1*_ (ESBL) [29]. However, the findings from the current study are unique as there are *E. coli* isolates which were simultaneously positive for colistin, extended spectrum beta-lactams and carbapenem resistance encoding genes.

In this study, 70.3% (19/27) *E. coli* isolates were colistin resistant and 55.56% (15/27) belonged to phylogroups A, whereas 33.3% (9/27) and 11.11% (3/27) were assigned to groups D and B1 respectively. These results are in agreement with studies by Johnson et al. [30] which reported that most of the *E coli* isolated from retailed chicken products predominantly belonged to groups A (32%) and D (25%). Studies conducted in United States [31] and Japan [32] have demonstrated that phylogenetic groups A and D are predominant among Avian Pathogenic *Escherichia coli* (APEC) isolates. Whereas Hiki et al*.* [33] reported that phylogenetic groups A and B1 comprised more than 80% of *E. coli* isolated from healthy broilers. Other studies also reported similar findings showing that isolates of phylogroup A were the most prevalent *E. coli* isolates in animal intestinal tract [34, 35]. The occurrence of group A and B1 in the raw chicken meat suggest that fecal contamination which may occur at any stage of processing could be a source of contamination [36]. Such possibilities may expose consumers to contaminated chicken meat and may increase the possibilities of acquiring foodborne illnesses especially if the meat is handled unhygienically and undercooked. The phylogenetic analysis of 148 APEC isolates from south China [37], revealed that group was A was the predominant (73%) phylotype followed by group D (14%). In this study, only one strain belonged to group B2 contrast to findings from other studies which reported that extraintestinal *E. coli* mostly belonged to group B2 [32]. Extraintestinal pathogenic *E. coli* are phylogenetically distinct from commensal and intestinal pathogenic *E. coli* and they mostly belong to group B2 [38]. Different studies report that the distribution of *E. coli* phylogroup are different based on several factors including environmental variations, species and health status of the host, the use of antimicrobials and sampling methods used [39, 40]. This might be helpful in clarifying the potential roles of the emergence of multidrug resistance bacteria causing human diseases and in ascertaining their animal sources.

As for AST results, all *E. coli* isolates from group A showed resistance against trimetoprim-sulfametoxazol, colistin and sulphonamide. *Escherichia coli* strains belonging to phylogroup A and B1 showed different susceptibility levels to colistin compared to *E. coli* isolates from phylogroup D. Among the 10 antibiotics used for the antibiogram, *E. coli* isolates showed moderate to high levels of resistance to 8 antibiotics. These results confirm the necessity of antimicrobial susceptibility tests for *E. coli* isolates in order to select appropriate antibiotics for treatment of diseases caused by these bacteria. In the current study, the most frequent resistant phenotype observed was against beta-lactam, tetracycline, sulphonamide and colistin which may imply the ineffectiveness of using these drugs in treating infections caused by the resistant *E. coli* strains identified in this and other similar studies. Tetracycline, sulfamethoxazole and chloramphenicol were the top three drugs recognized as the most common drugs to which resistance develops [41]. Evidently *E. coli* strains have breached the last group of antibiotics, colistin which has been used mainly when bacterial infections do not respond to other drugs. Our results show that the prevalence of colistin resistance is 8.64% (19/220) and this finding is relatively higher. This relatively high level of resistance reported in the current study may be attributed to the possibility of widespread use of this low-cost antimicrobial drug as a growth promoter in the livestock industry locally in the previous years.

In this study, the widespread *E. coli* strains belonging to the sequence types ST410, ST155, ST2179 were detected. Among this, *E. coli* ST410 has been widely reported as an extraintestinal pathogen associated with various antimicrobial resistance determinants, including ESBLs, pAmpCs, carbapenemases, and colistin resistance genes worldwide. The strain represents a globally distributed lineage that has been isolated in Europe, North America, South America, Asia, and Africa and was reported to have the ability to persist within a host for long period of time. Recent studies indicate that *E. coli* ST410 is another successful pandemic extraintestinal pathogenic *E. coli* (ExPEC) lineage similar to ST131 [42]. Likewise, *E. coli* ST155 has been widely reported in several countries and regions and have been suggested as among the *E. coli* clones that are shared between poultry and humans [43]. Whereas the multi-drug resistant *E. coli* ST2179 has been isolated from horses in Brazil and was characterized as a causative agent for extra-intestinal infections with possible zoonotic transmission between animals and humans [44].

Phylotyping analysis revealed that the most prevalent multiple drug resistant strain belonged to phylogroups A and B1. Whereas phylogroups D and F *E. coli* isolates were more susceptible to antibiotics than isolates belonging to group A and B1. Several studies have reported similar significant results indicating that although being more virulent, the isolates of phylogroup D showed more susceptibility to antibiotics [45, 46]. This explains that strains belonging to phylogroups A and B1 might carry more resistance properties than the strains belonging to phylogroups D and F. Based on the current findings, it is concluded that raw chicken meat in the study area could act as reservoir for such resistant strains. As it has been reported from different studies, a major contributing factor for the development of selective antimicrobial resistance in bacteria are irrational use of antibiotics and poor animal husbandry practices [47]. These factors are typical of intensive poultry farming in most developing countries and may explain the high prevalence of resistance in *E. coli* from poultry meat [48].

## Conclusions

In conclusion, the current study showed that *E. coli* from live chicken and chicken meat harbor multiple antibiotic resistance genes including carbapenem, colistin and extended beta lactam resistance genes. Notably, an isolate from cloacal swab was positive for carbapenemases genes (*bla*_*NDM*_ and *bla*_*OXA-48*_), colistin resistance encoding gene (*mcr*-1) and extended spectrum beta-lactamase encoding gene (*bla*_*TEM-52*_). Whereas five other *E. coli* isolates were positive for *mcr*-1, *bla*_*TEM-52*_, and *bla*_*IMP*_/*bla*_*OXA-48*_ genes. These traits underscore the importance and urgency to encounter the emergence and spread of these resistant bacteria that may pose serious public health risks. It is imperative to enforce strict compliance to antibiotics stewardship and infection control practices in medical, veterinary practices, in agriculture and other stake holders within the frame of one health. Furthermore, livestock farmers need to be educated in improving farming practices that can reduce the carriage of antibiotic resistance genes and thereby minimize the likelihood of horizontal gene transfers of these antimicrobial resistance genes to other microbes in the food chain.

## Data Availability

All data generated or analyzed during the present study are included in this manuscript.
